# Avicularin alleviates acute liver failure by regulation of the TLR4/MyD88/NF‐κB and Nrf2/HO‐1/GPX4 pathways to reduce inflammation and ferroptosis

**DOI:** 10.1111/jcmm.17905

**Published:** 2023-08-29

**Authors:** Pei Shi, Wentao Zhu, Jiwei Fu, An Liang, Ting Zheng, Zhilong Wen, Xincheng Wu, Yuchen Peng, Songsong Yuan, Xiaoping Wu

**Affiliations:** ^1^ Department of Infectious Diseases The First Affiliated Hospital of Nanchang University Nanchang China; ^2^ Medical Innovation Center The First Affiliated Hospital of Nanchang University Nanchang China

**Keywords:** acute liver failure, avicularin, ferroptosis, inflammation, Nrf2/HO‐1/GPX4, TLR4/MyD88/NF‐κB

## Abstract

Acute liver failure (ALF) is an inflammation‐mediated hepatocyte death process associated with ferroptosis. Avicularin (AL), a Chinese herbal medicine, exerts anti‐inflammatory and antioxidative effects. However, the protective effect of AL and the mechanism on ALF have not been reported. Our in vivo results suggest that AL significantly alleviated lipopolysaccharide (LPS)/D‐galactosamine (D‐GalN)‐induced hepatic pathological injury, liver enzymes, inflammatory cytokines, reactive oxygen species and iron levels and increased the antioxidant enzyme activities (malondialdehyde and glutathione). Our further in vitro experiments demonstrated that AL suppressed inflammatory response in LPS‐stimulated RAW 264.7 cells via blocking the toll‐like receptor 4 (TLR4)/myeloid differentiation protein‐88 (MyD88)/nuclear factor kappa B (NF‐κB) pathway. Moreover, AL attenuated ferroptosis in D‐GalN‐induced HepG2 cells by activating the nuclear factor erythroid 2‐related factor 2 (Nrf2)/heme oxygenase 1 (HO‐1)/glutathione peroxidase 4 (GPX4) pathway. Therefore, AL can alleviate inflammatory response and ferroptosis in LPS/D‐GalN‐induced ALF, and its protective effects are associated with blocking TLR4/MyD88/NF‐κB pathway and activating Nrf2/HO‐1/GPX4 pathway. Moreover, AL is a promising therapeutic option for ALF and should be clinically explored.

## 1 INTRODUCTION

Acute liver failure (ALF) is a complex and multifactorial acute liver dysfunction. It is characterized by deteriorating liver function and rapidly evolving multiple organ failure.^1^ The aetiology of ALF is multiple, including hepatotoxic agents, hepatitis virus infection, alcohol, ischemic and autoimmune hepatitis.^2^ Systemic inflammation and hepatocyte death are two important factors involving in the pathophysiology in ALF.^3^, ^4^ Studies have shown that regulation of inflammation and ferroptosis are effective strategies for preventing and blocking ALF.^5^, ^6^, ^7^

Inflammation is the core pathogenesis of ALF.^8^ An essential feature of ALF is systemic inflammatory response syndrome (SIRS),^9^, ^10^ which is driven by the release of pro‐inflammatory mediators by immune cells. SIRS is associated closely with organ failure and mortality in ALF.^11^ The main causes of death in ALF are excessive activation of SIRS complicated by immune dysfunction, septic shock and multiorgan failure.^12^, ^13^ Lipopolysaccharide (LPS), is also called endotoxin, which can induce inflammatory mediators release, is the major factor that leads to liver injury.^14^ LPS/D‐galactosamine (D‐GalN) is the classical animal model of endotoxemia‐induced ALF.^15^ Inhibition of pro‐inflammatory cytokines production and their release could alleviate hepatic inflammation and even systemic inflammation, protecting from liver damage.^16^, ^17^

Ferroptosis is a recently discovered pattern of cell death caused by iron‐dependent oxidative damage.^18^ Compared to the normal liver, iron and lipid reactive oxygen species (ROS) in the diseased liver were increased, suggesting that ferroptosis may play an important role in liver. There is increasing evidence that ferroptosis may be associated with liver cell death in ALF.^7^, ^19^ Therefore, regulating ferroptosis may be a novel treatment strategy for ALF.

Ferroptosis is characterized by the accumulation of lipid peroxides and ROS.^20^ Biochemically, the intracellular glutamate/cystine antitransport system (Xc‐system) is inhibited, glutathione (GSH) is depleted and glutathione peroxidase 4 (GPX4) activity is reduced, leading to the massive generation of lipid peroxide and ROS, and promotes ferroptosis.^21^ The Xc‐system is a heterodimer composed of light chain SLC7A11 (xCT) and heavy chain SLC3A2 (4F2hc), which can transport glutamate to the outside of the cell and simultaneously transport cystine into the cell, and cystine participates in the synthesis of GSH.^22^ GPX4 is the main scavenger of intracellular lipid peroxides, which can convert GSH to oxidized glutathione while reducing toxic peroxides, so GPX4 is a master regulator of ferroptosis.^23^

**FIGURE 1:**
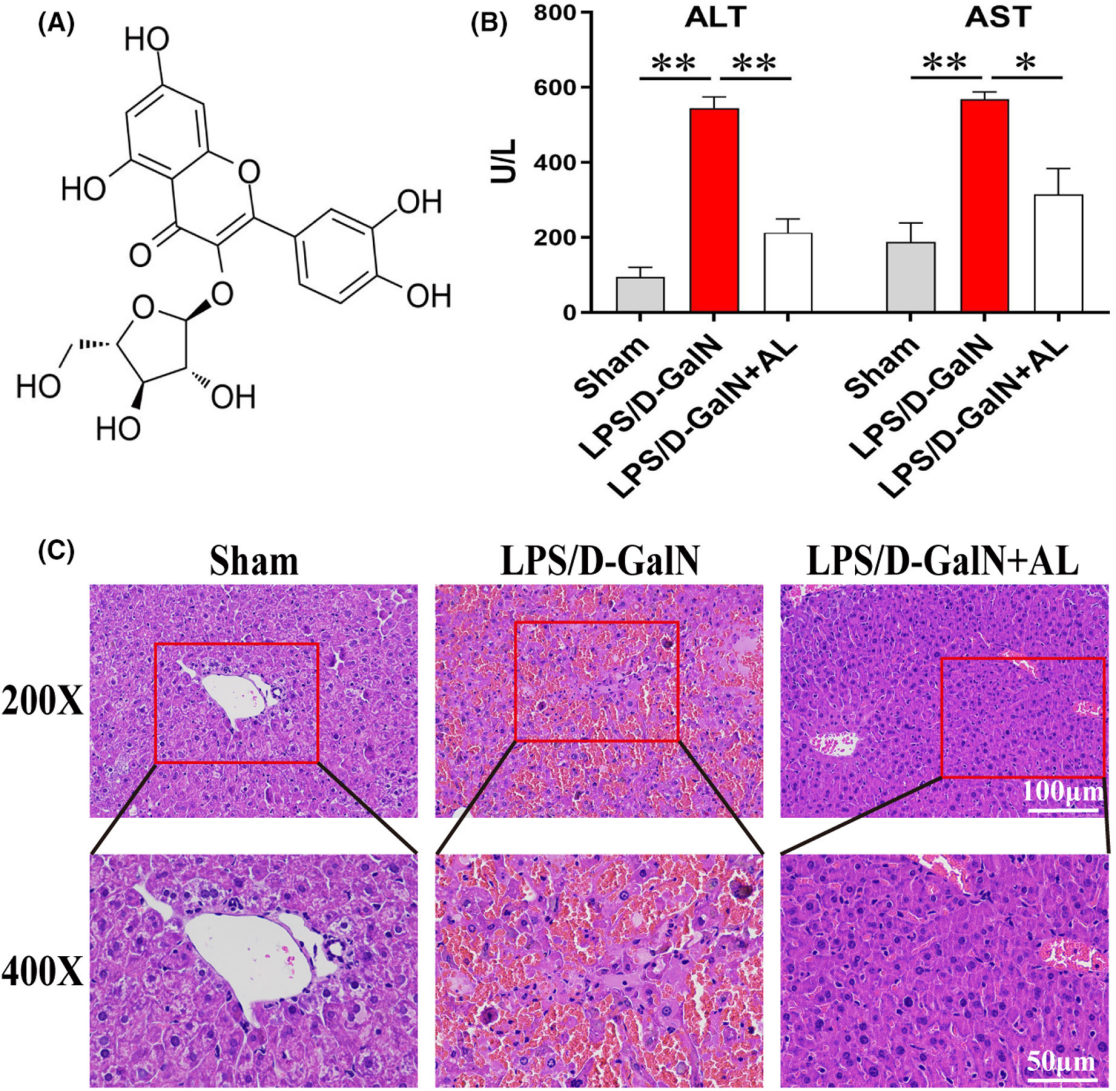
Avicularin alleviated LPS/D‐GalN‐induced acute liver failure in mice. (A) The chemical structure of avicularin (AL). (B) ALT and AST levels in ALF mice pretreatment with AL (50 mg/kg). Data are presented as the mean ± SD from three independent experiments. (C) Representative image of histopathological examination of liver tissue by H&E staining. Data are presented as the mean ± SD from three independent experiments. *p < 0.05 and **p < 0.01.

Synthetic drugs have some disadvantages, such as easy side effects, drug resistance and environmental pollution. Additional, lacking of standardization and randomized clinical trials restricted its clinical promotion.^24^ Recently, natural Chinese herbal medicine has been extensively found for its anti‐inflammatory and antioxidative advantages, may be alleviate inflammatory cytokine storm and hepatocyte death in ALF. Avicularin (AL; Figure 1A), quercetin‐3‐α‐L‐arabinofuranoside, is a flavonoid widely existing in dicotyledon plants. It has extensive pharmacological activities including anti‐inflammatory, antioxidative, anti‐allergic and anti‐tumour.^25^, ^26^, ^27^ However, the protective effect of AL on ALF and its underlying mechanism have not been reported. In this study, we found that AL can significantly improve LPS/D‐GalN‐induced ALF in vivo also used LPS‐stimulated RAW 264.7 cells and D‐GalN‐induced HepG2 cells to build inflammatory model and hepatocyte injury model to study the anti‐inflammatory and anti‐ferroptosis effects of AL and their molecular mechanisms.

## 2 MATERIALS AND METHODS

### 2.1 Chemicals

AL was provided by Shenzhen Zhenqiang Biotechnology Corporation with a purity >98%. LPS, D‐GalN, Fer‐1, TAK242, PDTC and ML385 were provided by Sigma‐Aldrich. The primary antibodies against inducible nitric oxide synthase (iNOS), cyclooxygenase‐2 (COX‐2), inhibitor kappa Bα (IκBα), phosphorylated‐IκBα (p‐IκBα), NF‐κB p65 (p65) and p‐NF‐κB p65 (p‐p65) were obtained from Abcam. Antibodies for erythroid 2‐related factor 2 (Nrf2), heme oxygenase 1 (HO‐1), glutathione peroxidase 4 (GPX4) and xCT/SLC7A11 were obtained from Cell Signalling Technology. Antibodies against TLR4, MYD88, β‐actin and GAPDH were purchased from Proteintech.

### 2.2 Animal model

Male C57BL/6 mice (20–25 g) from Tianqin Biotechnology were randomized (*n* = 18, 6 per group): control group, LPS/D‐GalN group, LPS/D‐GalN + AL (50 mg/kg) group. LPS (10 μg/kg) and D‐GalN (800 mg/kg) were injected intraperitoneally into mice to induce ALF. For pretreatment, AL (50 mg/kg) was intragastrically administered to the mice for 3 days before LPS/D‐GalN administration. To monitor the liver injury, the mice were killed at 1, 3 and 5 h after the LPS/D‐GalN injection. Blood and liver samples were taken for subsequent analysis. The dose of AL was referenced to our preliminary experiments and previous literatures. The test was approved and supervised by the Laboratory Animal Committee of Nanchang University.

### 2.3 Biochemical assay for AST/ALT

Blood samples were collected from the retroorbital venous plexus and centrifugation at 3500 rpm for 10 min. Serum ALT and AST were detected by Hitachi 7600 automatic biochemical analyzer.

### 2.4 Histopathological analysis

A portion of liver tissue were placed in the fixing solution containing 4% paraformaldehyde (PFA). After 24 h, liver tissue was embedded in paraffin and were then stained with haematoxylin and eosin (H&E). The images were collected and analysed under an optical microscope.

### 2.5 Cell culture

The RAW 264.7 cells and HepG2 cells, purchased from Pricella, were cultured with high glucose DMEM medium with 10% FBS (Gibco). The RAW 264.7 cells were pretreated overnight with the corresponding concentration of AL and then stimulated with LPS (1 μg/mL) for 24 h. The HepG2 cells were pretreated overnight with the corresponding concentration of AL and then stimulated with D‐GalN (50 mM) for 6 h.

### 2.6 Molecular docking analysis

TLR4 (PDB ID: 4G8A) and Nrf2 (PDB ID: 2FLU) were selected as docking molecules. Three‐dimensional structure of protein was acquired from PDB database (https://www.rcsb.org/). Structural modification of protein was conducted using PyMOL (version 1.7.2.1) and protein–ligand docking analysis was completed by AutoDockTools (version 1.5.6). The docking conformation was finally visualized in PyMOL (version 1.7.2.1).

### 2.7 Real‐time quantitative PCR (RT‐qPCR)

Total RNA, extracted by TRIzol reagent (Invitrogen), was used to conduct RT‐qPCR.^28^ GAPDH was used as the reference gene and 2^−ΔΔCt^ method was used to analyse relative transcript levels. Sequence of primers used for RT‐qPCR are shown in Table 1.

**TABLE 1:** Sequence of primers used for RT‐qPCR assay.

Genes	Forward	Reverse
TNF‐α	CTCTTCTGTCTACTGAACTTCGGG	GGTGGTTTGTGAGTGTGAGGGT
IL‐1β	GCATCCAGCTTCAAATCTCGC	CAAAGTTGTCTGATTCCAGGTCTC
IL‐6	CTCCCAACAGACCTGTCTATAC	CCATTGCACAACTCTTTTCTCA
iNOS	AGCTCGGGTTGAAGTGGTATG	CACAGCCACATTGATCTCCG
COX‐2	GAAATATCAGGTCATTGGTGGAGA	ATGCTCCTGCTTGAGTATGTCG
GAPDH	CCTCGTCCCGTAGACAAAATG	TGAGGTCAATGAAGGGGTCGT

### 2.8 Western blot assay

Proteins were extracted by RIPA buffer (Beyotime). Samples were separated by SDS‐PAGE and then transferred to PVDF membranes (EMD Millipore). The membranes were blocked with skimmed milk powder, followed by incubated with primary antibodies and secondary antibodies. The bands were formed with ECL reagents (Beyotime) and analysed with ImageJ 6.0 software.

### 2.9 Immunofluorescence

After treatment, cells were fixed with PFA for 15 min and permeated with 0.3% Triton X‐100 for 20 min. Cells were blocked with goat serum for 1 h and were incubated with primary antibodies overnight at 4°C. Cells were washed with PBS and subsequently incubated with secondary antibodies at 37°C for 1 h. Finally, DAPI was used to immerse the cell slides for 5–8 min. The stained cells were visualized by a fluorescence microscope (ZEISS).

### 2.10 Malondialdehyde, glutathione and iron assay

The malondialdehyde (MDA), glutathione (GSH) and iron contents in cell or tissue extracts were detected using corresponding assay kits (Nanjing Jiancheng or Elabscience), respectively.

## 3 ROS ASSAY

ROS levels in liver tissue were measured using ROS Assay Kit (Elabscience). ROS activities of cells were assessed using a fluorescent probe (DCFH‐DA) (Keygen) and was analysed via a flow cytometer (NovoCyte).

### 3.1 Mitochondrial membrane potential (MMP) measurement

A mitochondrial membrane potential detection kit (JC‐1) (BestBio) was used to detect the changes in MMP. HepG2 cells were stained with JC‐1 for 20 min via incubation in the working solution, and then a flow cytometer (NovoCyte) was used to observe the relative fluorescence intensity ratio of red and green.

### 3.2 Statistical analysis

All values were presented as the means ± SEM. One‐way analysis of variance (anova) was used for multiple comparisons. GraphPad Prism 8.0 Software was used for data analyses. *p* < 0.05 was regarded as a statistical significance.

## 4 RESULTS

### 4.1 Effect of avicularin on LPS/D‐GalN‐induced acute liver failure

To investigate the effect of AL on LPS/D‐GalN‐induced ALF, we first evaluated the protective effect of AL on hepatotoxicity and found that LPS/D‐GalN significantly increased serum ALT and AST levels. However, AL (50 mg/kg) significantly reduced these increases (Figure 1B). Next, we examined the histopathology of liver tissue. As shown in Figure 1C, LPS/D‐GalN resulted in significant pathological damage characterized by structural destruction, extensive bleeding, necrosis and neutrophil infiltration. However, AL (50 mg/kg) significantly attenuated these morphological damage, only focal hepatocyte death was observed. Proinflammatory cytokines are considered to be key potent contributors to hepatocyte necrosis and organ failure.^29^ The results of recovery of liver tissues by AL may be related to the reduction of secondary stress injury and inflammation in the liver.

### 4.2 Avicularin reduced inflammatory response in mice

**FIGURE 2:**
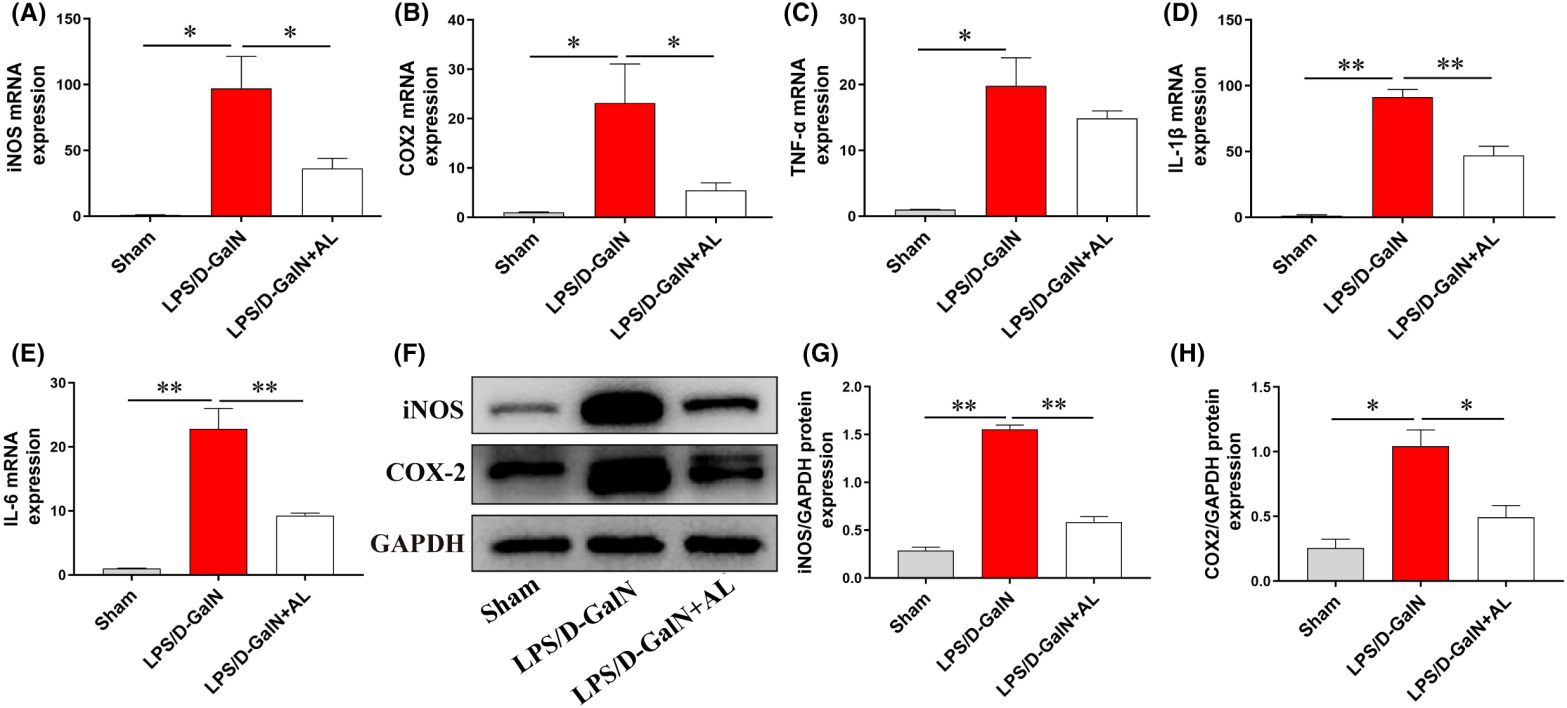
Avicularin reduced inflammatory response in mice. (A–E) The transcriptional levels of proinflammatory mediators iNOS, COX‐2, TNF‐α, IL‐1β and IL‐6 mRNA in the liver tissue. (F–H) The protein expression levels of iNOS and COX‐2 in the liver tissue. Data are presented as the mean ± SD from three independent experiments. *p < 0.05 and **p < 0.01.

To explore the underlying mechanism, we first detected the expression of inflammation because AL has anti‐inflammatory activity. The mRNA and protein levels of pro‐inflammatory mediators (iNOS, COX‐2, IL‐6, TNF‐α and IL‐1β) in liver tissue were dramatically increased in LPS/D‐GalN treatment group. Administration of AL inhibited the release of inflammatory cytokines induced by LPS/D‐GalN (Figure 2A‐H). A good protective effect of AL against ALF was observed. These results suggested that AL protected against ALF through its anti‐inflammatory activity.

### 4.3 Avicularin inhibited liver lipid peroxidation in mice

**FIGURE 3:**
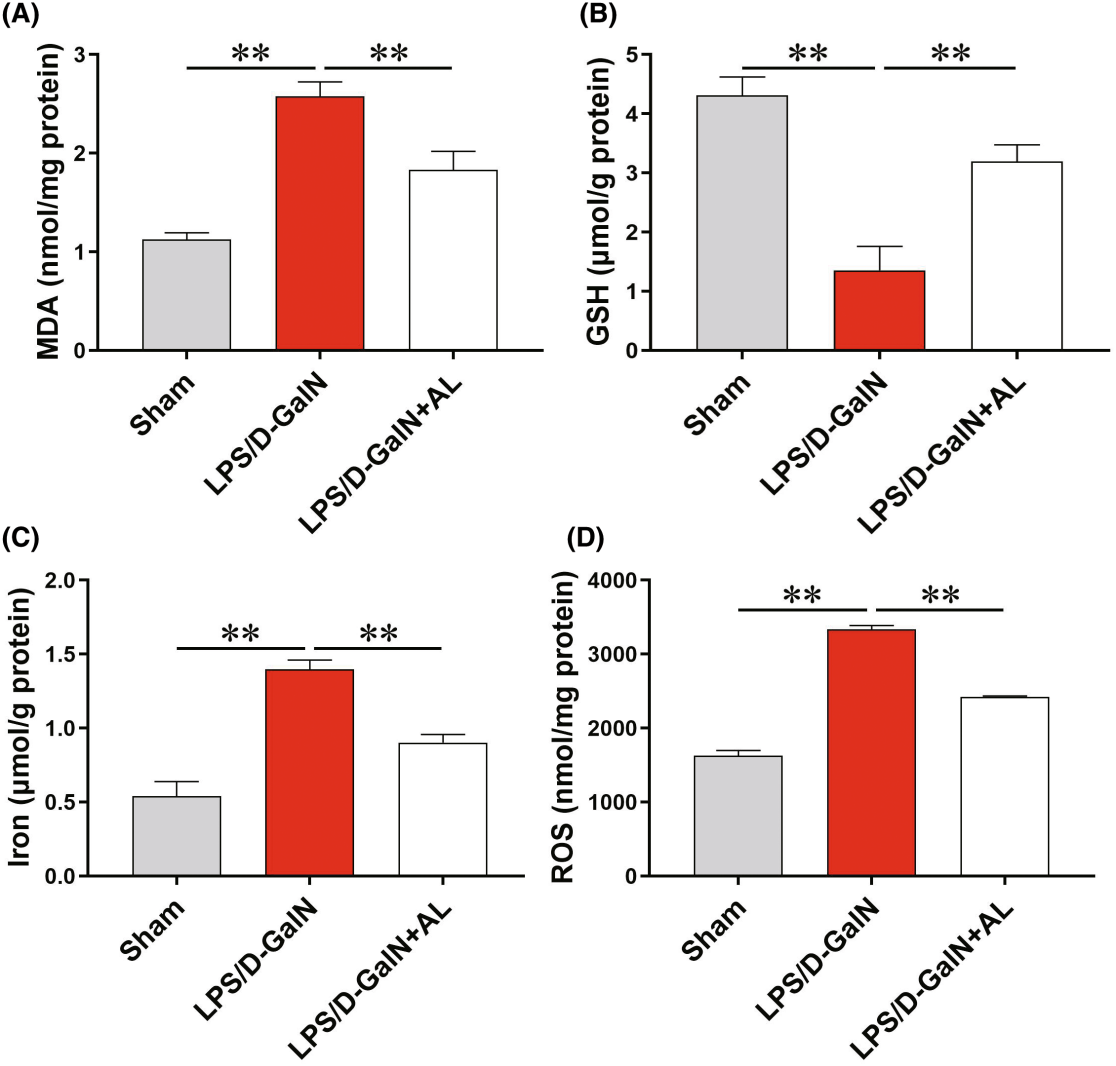
Avicularin inhibited liver lipid peroxidation in mice. (A) Avicularin (AL) significantly decreased the level of MDA. (B) AL significantly increased the GSH content. (C) Liver iron content significantly reduced after AL administration. (D) AL efficiently reversed the increase of ROS level. Data are presented as the mean ± SD from three independent experiments. **p < 0.01.

Lipid peroxidation is the essence of ferroptosis, to examine whether ferroptosis is involved or not in ALF, we first detected MDA, the lipid peroxidation indicator. As shown in Figure 3A, LPS/D‐GalN induced an increase in MDA context in the liver tissue, while AL efficiently blocked the increase. In addition, AL reversed the decrease of GSH content (Figure 3B) and inhibited the production of ROS and Fe, two central links of ferroptosis (Figure 3C,D). These results suggested that AL might exert its protective effect partly due to the inhibition of ferroptosis in LPS/D‐GalN‐induced ALF.

### 4.4 RAW 264.7 cells were activated by LPS in a time‐dependent manner

**FIGURE 4:**
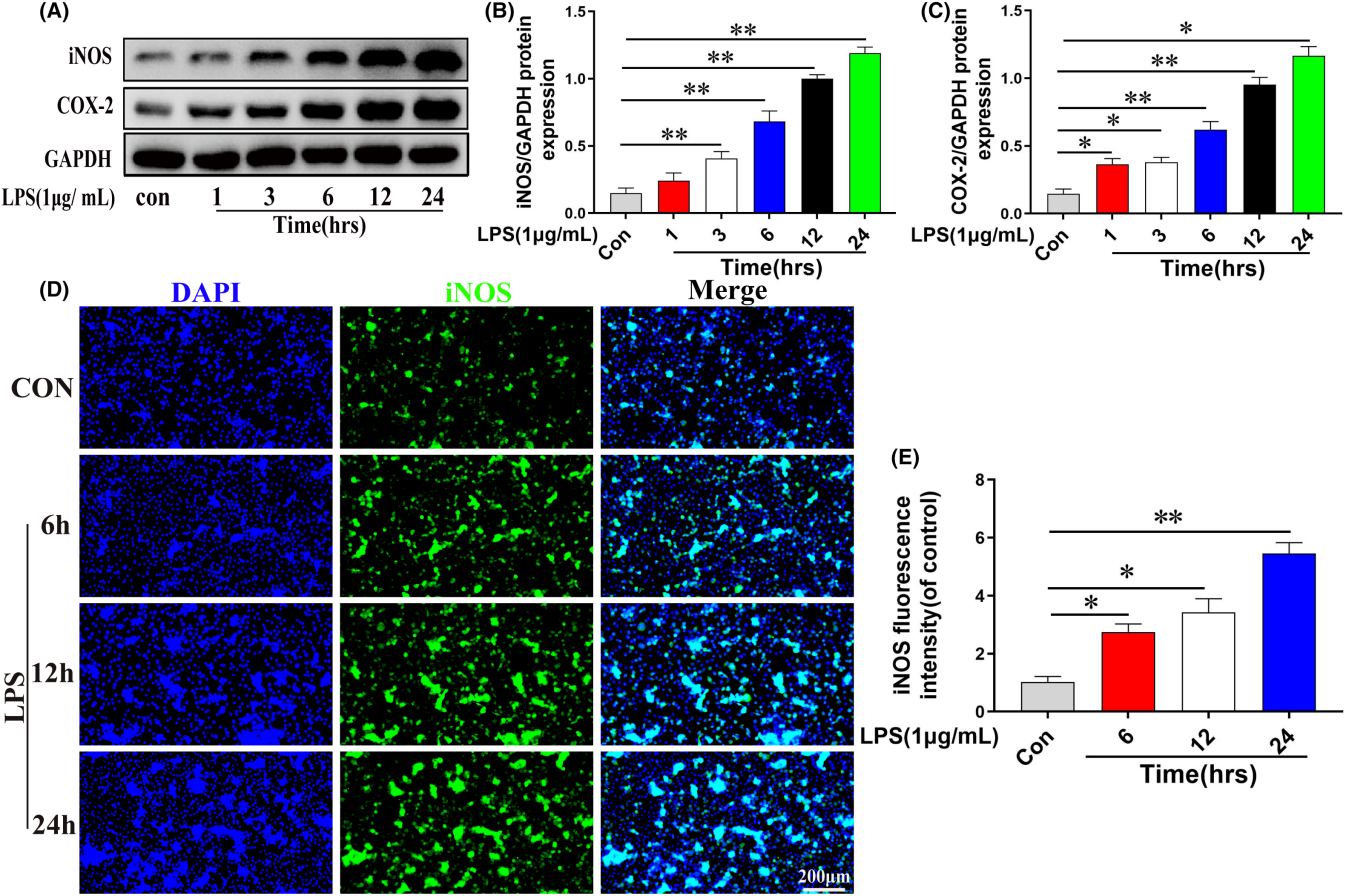
RAW 264.7 cells were activated by LPS in a time‐dependent manner. (A–C) iNOS and COX‐2 were determined by western blotting in RAW 264.7 cells treated with 1 μg/mL LPS for 0, 1, 3, 6, 12 and 24 h. (D–E) iNOS were determined by immunofluorescence staining in RAW 264.7 cells treated with 1 μg/mL LPS for 0, 6, 12 and 24 h. Data are presented as the mean ± SD from three independent experiments. *p < 0.05 and **p < 0.01.

To construct an in vitro model of inflammation, LPS was added to RAW 264.7 cells for 0–24 h at a concentration of 1 μg/mL. The protein expression levels of iNOS and COX‐2 in RAW 264.7 cells were time‐dependently increased after LPS treatment (*p* < 0.05; Figure 4A–C). Figure 4D,E showed agreement between immunofluorescence staining and western blotting for iNOS (*p* < 0.05). Therefore, for the stimulation condition, we selected 1 μg/mL as the dosage and 24 h as the stimulation period.

### 4.5 Effects of AL on activity of RAW 264.7 cells and LPS‐induced inflammatory response

**FIGURE 5:**
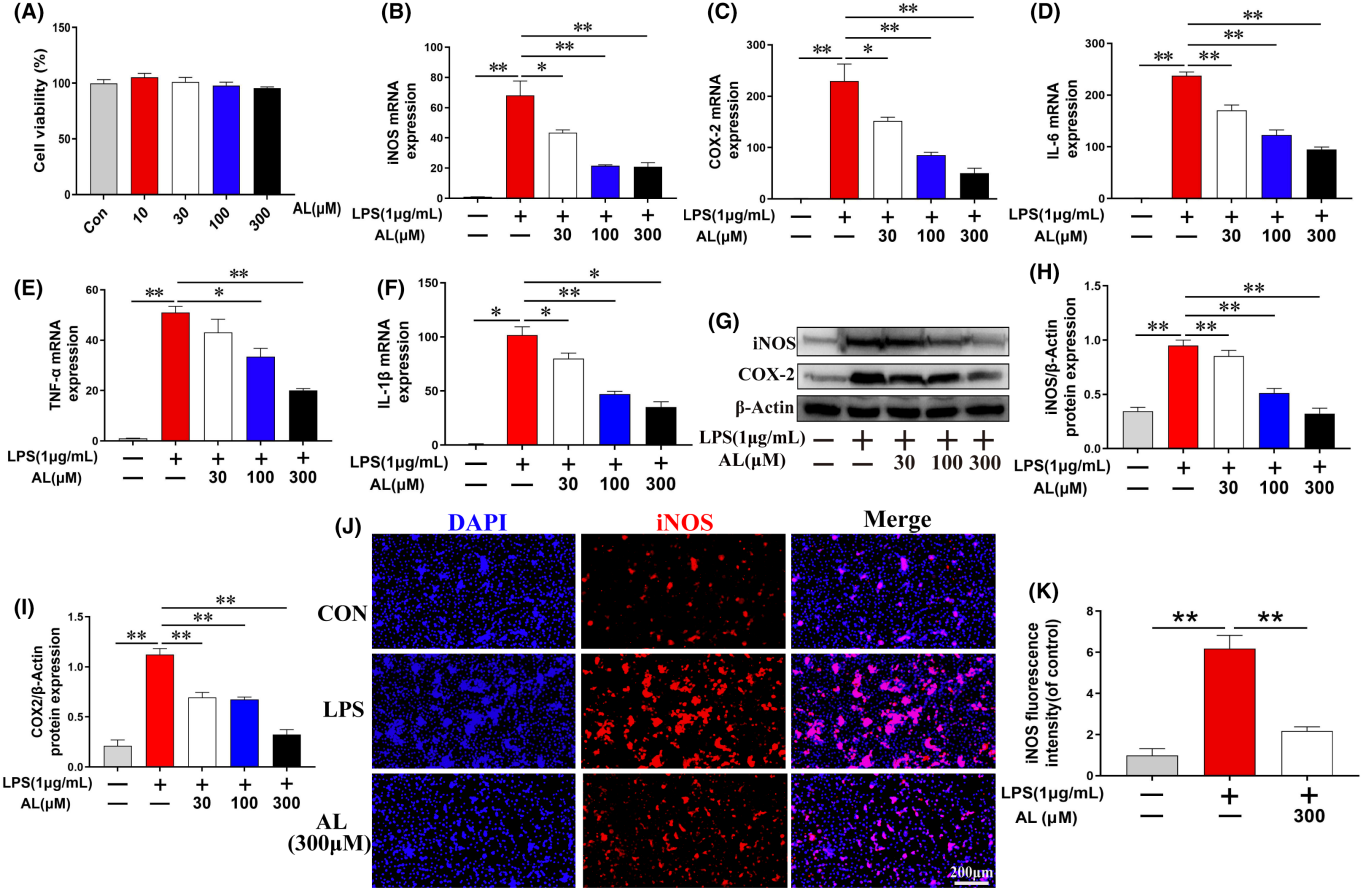
Avicularin inhibited the activation of inflammatory response in LPS‐stimulated RAW 264.7 cells. (A) The CCK‐8 assay was applied to assess the viability of RAW 264.7 cells treated with avicularin (AL) at different concentrations for 24 h. (B–F) iNOS, COX‐2, IL‐6, TNF‐α and IL‐1β mRNA levels were determined by quantitative real‐time reverse transcription‐polymerase chain reaction in RAW 264.7 cells treated with AL and LPS (1 μg/mL) for 24 h. (G–I) iNOS and COX‐2 were determined by western blotting in RAW 264.7 cells treated with AL and LPS (1 μg/mL) for 24 h. (J–K) iNOS were determined by immunofluorescence staining in RAW 264.7 cells treated with AL and LPS (1 μg/mL) for 24 h. Data are presented as the mean ± SD from three independent experiments. *p < 0.05 and **p < 0.01.

To determine the appropriate dosage of AL, different concentrations of AL were applied to RAW 264.7 cells. The cytotoxic effects of AL on RAW 264.7 cells were assessed using the Cell Counting Kit‐8 (CCK8). The results indicated that no cytotoxicity occurred at doses of 10–300 μM (Figure 5A). Thus, 30, 100 and 300 μM of AL was used in the following in vitro experiments.

We next studied the effect of AL on LPS‐induced inflammatory response. RAW 264.7 cells were pretreated overnight with 30, 100 and 300 μM AL and then treated for 24 h with or without 1 μg/mL LPS. LPS markedly increased the accumulation of iNOS, COX‐2, IL‐6, TNF‐α and IL‐1β, indicating that RAW 264.7 cells were in an inflammatory state. Treatment with AL reversed these changes in a dose‐dependent fashion (Figure 5B–I). The immunofluorescence staining results were consistent with western blot results (Figure 5J,K). These data suggested the protective effects of AL against ALF may be due to the decreased inflammatory cytokine levels.

### 4.6 Effect of avicularin on LPS‐induced TLR4/MyD88/NF‐κB pathway activation

**FIGURE 6:**
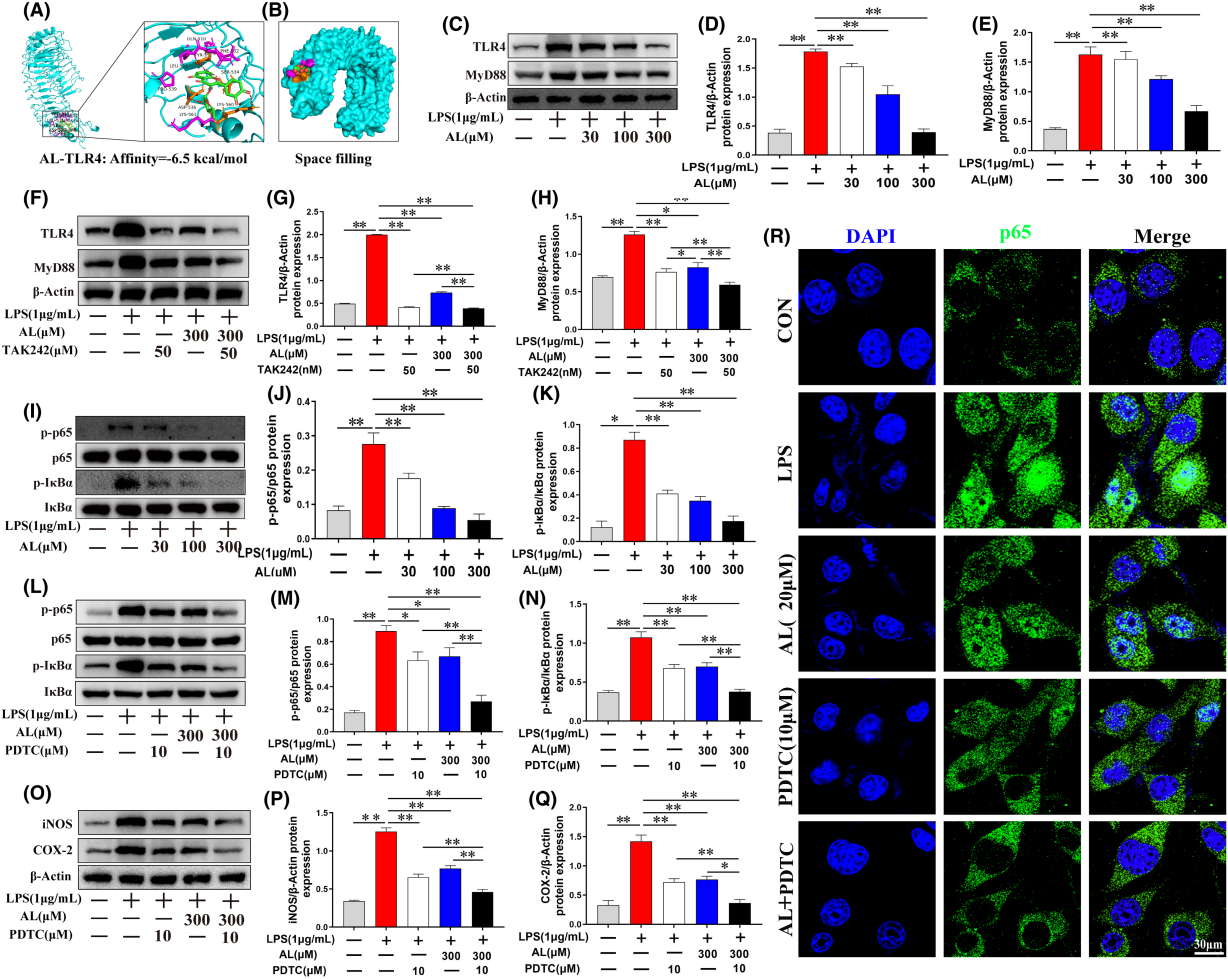
Avicularin blocked TLR4/MyD88/NF‐κB pathway in LPS‐stimulated RAW 264.7 cells. (A) Molecular docking of avicularin (AL) small molecule and TLR4 protein was conducted using Autodock vina. (B) Space filling model showing AL binding in the TLR4‐binding pocket. (C–H) Levels of TLR4 and MyD88 were determined by western blotting in each group of RAW 264.7 cells. (I–N) Levels of phosphorylated and total p65 and IκBα were determined by western blotting in each group of RAW 264.7 cells. (O–Q) iNOS and COX‐2 were determined by western blotting in each group of RAW 264.7 cells. (R)The translocation of the NF‐κB p65 were determined by immunofluorescence staining. Data are presented as the mean ± SD from three independent experiments. *p < 0.05 and **p < 0.01.

The activity of TLR4‐mediated MyD88 pathway is correlated with ALF and inflammation.^13^, ^30^ To further discover the mechanism underlying anti‐inflammatory effect, we measured the effect of AL on TLR4‐MyD88 signalling. Molecule docking study was done to examine the binding affinity and mode of interaction between TLR4 and AL. As shown in Figure 6A, spatial structure and hydrogen bonds after combination were shown in a ribbon model. A space‐filling model (Figure 6B) was performed to directly demonstrate the coverage of AL in the related protein structures. The analysis of molecule docking suggested that AL formed some good connections with the TLR4‐binding sites, with a high affinity of −6.5 kcal/mol. The experiments indicated that compared to the untreated cells, protein expression of TLR4 and MyD88 were greatly increased in LPS‐treated cells. Whereas AL treatment dose‐dependently reduced these protein levels (*p* < 0.05; Figure 6C–E). To further elucidate whether AL reduced the secretion of pro‐inflammatory mediators in macrophage via the TLR4/MyD88 pathway, RAW 264.7 cells were treated with TAK242, a TLR4 pathway inhibitor. As shown in Figure 6F–H, compared with TAK242 or AL treatment alone, co‐treatment with TAK242 and AL had stronger inhibitory effects on the protein expression of TLR4 and MyD88. These results demonstrated that AL exerted anti‐inflammatory effects through modulation of the TLR4/MyD88 pathway.

Furthermore, inflammatory reaction mediated by the TLR4‐MyD88 pathway closely associates with other numerous signalling pathways, including NF‐κB pathway, which has been proved to induce inflammatory cytokines production.^31^, ^32^ We also assessed the action of AL on the activation of the NF‐κB pathway in vitro. Compared with the untreated cells, LPS treatment augmented phosphorylation expression of IκBα and p65, however, AL therapy significantly inhibited this effect, as determined by western blot (*p* < 0.05; Figure 6I–K). PDTC, a NF‐κB pathway inhibitor, was applied to explore the action of NF‐κB pathway on AL‐mediated anti‐inflammatory effect. The results showed that the combination of PDTC and AL inhibited expression of p‐IκBα, p‐p65, iNOS and COX‐2 more than that of PDTC or AL treatment alone (*p* < 0.05; Figure 6L–Q). These results were confirmed by immunofluorescence, which indicated that co‐treatment with PDTC and AL markedly suppressed LPS stimulation‐induced translocation of p65 (Figure 6R). All these results indicated that AL mitigated inflammation through inhibition of TLR4 mediated NF‐κB activation.

### 4.7 Avicularin inhibited ferroptosis in D‐GalN‐induced HepG2 cells

**FIGURE 7:**
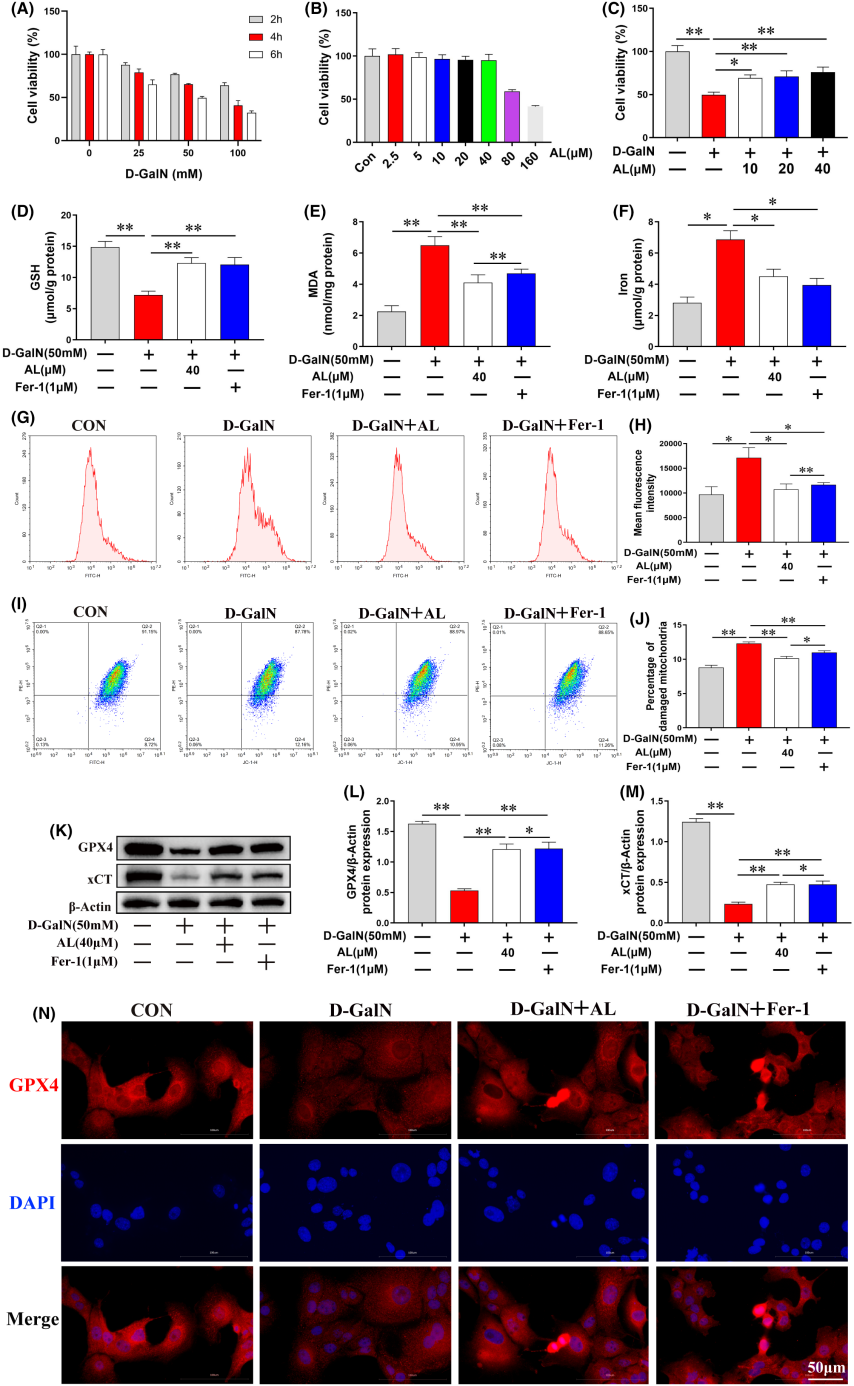
Avicularin (AL) inhibited ferroptosis in D‐GalN‐induced HepG2 cells. (A) HepG2 cells were treated with D‐GalN. Cell viability was measured by CCK8 assay. (B) Effect of AL on the viability of HepG2 cells. (C) HepG2 cells were treated with increasing concentrations of AL (10 μM, 20 μM and 40 μM) for 24 h, then treated with D‐GalN (50 mM) for 24 h. Cell viability was measured by CCK8 assay. (D) D‐GalN significantly reduced the GSH content, while AL administration increased the GSH content. (E) The test of MDA content in the cells. (F) AL decreased the intracellular iron content. (G, H) The flow cytometry showed that D‐GalN significantly promoted the production of ROS, while AL significantly reduced the ROS production. (I, J) JC‐1 staining of HepG2 cells were measured by flowcytometry. Apoptotic cells were identified by JC‐1 staining (green), and non‐apoptotic cells were identified by JC‐1 staining (red). (K–M) Western blot showed that AL significantly promoted the expression of GPX4 and xCT. (N) GPX4 were determined by immunofluorescence staining in each group of HepG2 cells. Data are presented as the mean ± SD from three independent experiments. *p < 0.05 and **p < 0.01.

To induce hepatocyte injury model in vitro, we treated HepG2 cells with D‐GalN (25, 50, 100 mM) for 2, 4, 6 h and then detected the cell activities with CCK8 assay. The results showed that D‐GalN‐induced cytotoxicity dose‐dependently and time‐dependently (Figure 7A). In the follow‐up experiment, we treated HepG2 cells with D‐GalN (50 mM) for 6 h. Next, we confirmed the inhibitory effect of AL on ferroptosis in vitro; the result of AL on activity of HepG2 cells showed that AL (10, 20 and 40 μM) inhibited HepG2 cell death caused by D‐GalN (50 mM) at 6 h (Figure 7B,C). Moreover, the ferroptosis inhibitor Fer‐1 (1 μM) was utilized to certify the effects of AL on hepatocyte ferroptosis. We detected the MDA, GSH, iron and ROS levels in the cells. Results found that both Fer‐1 and AL somewhat alleviated iron overload, reduced lipid peroxidation (MDA, ROS) and prevented the depletion of GSH in D‐GalN‐induced HepG2 cells (Figure 7D–H). To detect the change of MMP, we employed fluorescent dye JC‐1, when mitochondrial damage occurs, the MMP is low, it will show green fluorescence. As shown in Figure 7I,J, AL dramatically reduced the ratio of mitochondrial‐damaged cells. The result of western blot showed that the protein expression of GPX4 and xCT increased strikingly by AL (40 μM) pretreatment (Figure 7K–M). GPX4 expression was further evaluated by immunofluorescence. The result was in line with protein expression of GPX4 (Figure 7N). It seems that anti‐ferroptosis is involved in the mechanism of AL against ALF.

### 4.8 Avicularin alleviated ferroptosis in D‐GalN‐induced HepG2 cells through activating the Nrf2/HO‐1/GPX4 pathway

**FIGURE 8:**
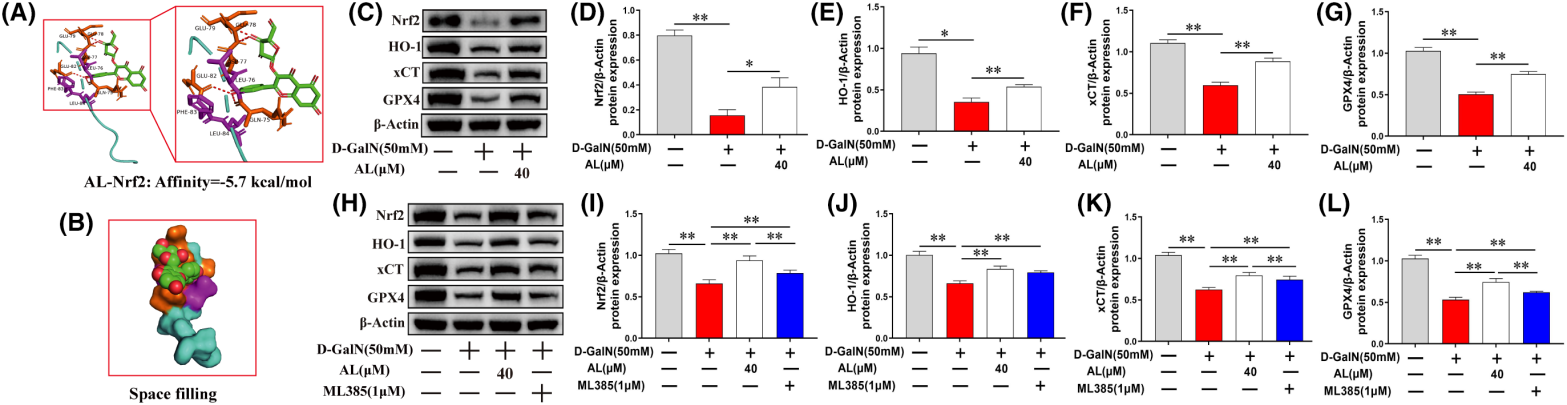
Avicularin (AL) activated Nrf2/HO‐1/GPX4 pathway in D‐GalN‐induced HepG2 cells. (A) Molecular docking of AL small molecule and Nrf2 protein was conducted using Autodock vina. (B) Space filling model showing AL binding in the Nrf2‐binding pocket. (C–G) Western blot showed that AL treatment significantly promoted the expression of Nrf2, HO‐1, GPX4 and xCT. (H–L) HepG2 cells were untreated or stimulated with D‐GalN, or pretreated with AL (40 μM) or AL (40 μM) plus ML385 (1 μM) for 1 h prior to treatment with D‐GalN. Then Nrf2, HO‐1, GPX4 and xCT protein levels were detected by western blot. Data are presented as the mean ± SD from three independent experiments. *p < 0.05 and **p < 0.01.

It has been reported that the activation of Nrf2/HO‐1 pathway might protect against ferroptosis in liver injury.^33^, ^34^ Molecular docking analysis indicated that AL small molecule interacted with the Nrf2 protein, with a binding energy of −5.7 kJ/mol (Figure 8A,B). To explore the molecular mechanism of AL on suppressing ferroptosis, we first measured the effects of AL on Nrf2 protein expression and its downregulated genes including HO‐1, GPX4 and xCT. Western blot results showed that D‐GalN obviously reduced the protein expression of Nrf2, HO‐1, GPX4 and xCT, which were rescued by AL (40 μM) (Figure 8C–G). Conversely, Nrf2 inhibitor ML385 (1 μM) significantly abrogated the influence of AL on Nrf2, HO‐1, GPX4 and xCT protein levels in D‐GalN‐treated cells (Figure 8H–L). These in vitro data support the idea that Nrf2/HO‐1/GPX4 pathway is involved in the anti‐ferroptosis activities of AL against ALF.

## 5 DISCUSSION

ALF is a severe acute and high‐mortality disease, liver transplantation is the only radical cure at present. However, medical treatment may strive for an effective treatment window for liver transplantation. Many studies have concentrated on the development of herbal medicines for the therapy of acute liver injury^6^, ^35^, ^36^, ^37^ such as tectorigenin^38^ and luteolin^39^ have been proved to exert protective effects against ALF. AL, which has the function of anti‐inflammatory, anti‐allergy, antioxidant, liver‐protective, anti‐tumour and other pharmacological activities,^25^, ^26^, ^27^, ^40^ is one of the main active ingredients of *Polygonum aviculare L*. To date, the protective effect of AL on ALF and its mechanism remain unclear. Our study indicated for the first time that AL attenuated LPS/D‐GalN‐induced ALF via inhibiting TLR4/MyD88/NF‐κB‐mediated inflammation activation and Nrf2/HO‐1/GPX4‐mediated ferroptosis.

For this purpose, we used a classic and mature mouse model of ALF induced by LPS/D‐GalN. ALF was characterized by elevated serum liver enzymes ALT and AST, liver structure disorder, infiltration of inflammatory cells, necrosis and bleeding, which was consistent with previous reports,^41^, ^42^ while AL pretreatment reduced liver enzyme activity and alleviated liver histopathological damage. Furthermore, AL pretreatment significantly reduced the levels of proinflammatory cytokines and a robust production of lipid peroxidation induced by LPS/D‐GalN.

TLR signalling plays a crucial role in cellular immunity.^43^ TLR4, a major signalling receptor of LPS, is closely linked to the pathogenesis and treatment of liver failure.^44^, ^45^ MyD88 is a critical downstream signal protein in response to TLR4 interaction with LPS, under the action of LPS stimulation, NF‐κB is activated by TLR4‐MyD88 signalling pathway.^46^ When NF‐κB pathway is activated, followed by IκBα is degraded and the phosphorylation of p65 is upregulated, which induce the translocation of p65 from the cytoplasm to the nucleus. This series of cascade reaction eventually results in inflammatory response activation and the release of inflammatory factors. During liver injury, innate immune cells release excessive amounts of inflammatory factors, induce inflammatory cascade reaction and inflammatory cytokine storm can be extremely deadly.^11^ It may be an effective method to protect against endotoxin‐induced ALF by inhibiting inflammation through regulating inflammatory signal transduction pathway.^47^, ^48^ Therefore, we investigated the underlying possible mechanism of AL and analysed the TLR4/MYD88/NF‐κB pathway. We found that LPS stimulation increased the expression of TLR4, MYD88, IκBα, p‐IκBα, p65, p‐p65 and regulated p65 nuclear translocation in RAW 264.7 cells, however, AL pretreatment inhibited the activation of TLR4/MyD88/NF‐κB pathway. The use of TAK242, TLR4 pathway inhibitor, or PDTC, an NF‐κB pathway inhibitor further suppressed release of inflammatory mediators, which was mediated by TLR4/MyD88/NF‐κB pathway. All these results suggested that the anti‐inflammatory effect of AL was partly the result of inhibiting TLR4/MyD88/NF‐κB pathway.

Inflammatory response cause ROS production, which plays an important role in the regulatory network of ferroptosis. Once the balance between the production and the clearance of lipid peroxidation is broken, ferroptosis will be triggered. Our study revealed that AL attenuated LPS/D‐GalN‐induced hepatotoxicity as effectively as Fer‐1 in vivo and in vitro. Next, we detected the effect of AL on liver antioxidant system and found that it strikingly mitigated LPS/D‐GalN‐induced lipid peroxidation, manifested by the levels of MDA, GSH and lipid ROS in liver tissues and hepatocytes. Moreover, AL also suppressed other ferroptosis markers including GPX4, xCT. The evidence fully supports our hypothesis that AL protects against ALF through inhibiting hepatocyte ferroptosis.

It is well established that Nrf2 is a crucial antioxidant transcription factor that regulates the expression of downstream genes including HO‐1, xCT and GPX4. Therefore, we observed the effects of AL on protein levels of xCT, GPX4, HO‐1 and Nrf2 in an in vitro hepatocyte injury model. As expected, D‐GalN significantly decreased protein levels of xCT, GPX4, HO‐1 and Nrf2, importantly, AL reversed those changes. These results showed that AL activates Nrf2 and its downstream signalling molecules. However, Nrf2 inhibitor ML385 abrogated the protective effects of AL on HO‐1, xCT and GPX4 activity, supporting that Nrf2 acts as an upstream mediator of HO‐1, xCT and GPX4 in D‐GalN‐induced HepG2 cells. Taken together, these results supported that AL activates Nrf2/HO‐1/GPX4 pathway in D‐GalN‐induced HepG2 cells.

**FIGURE 9:**
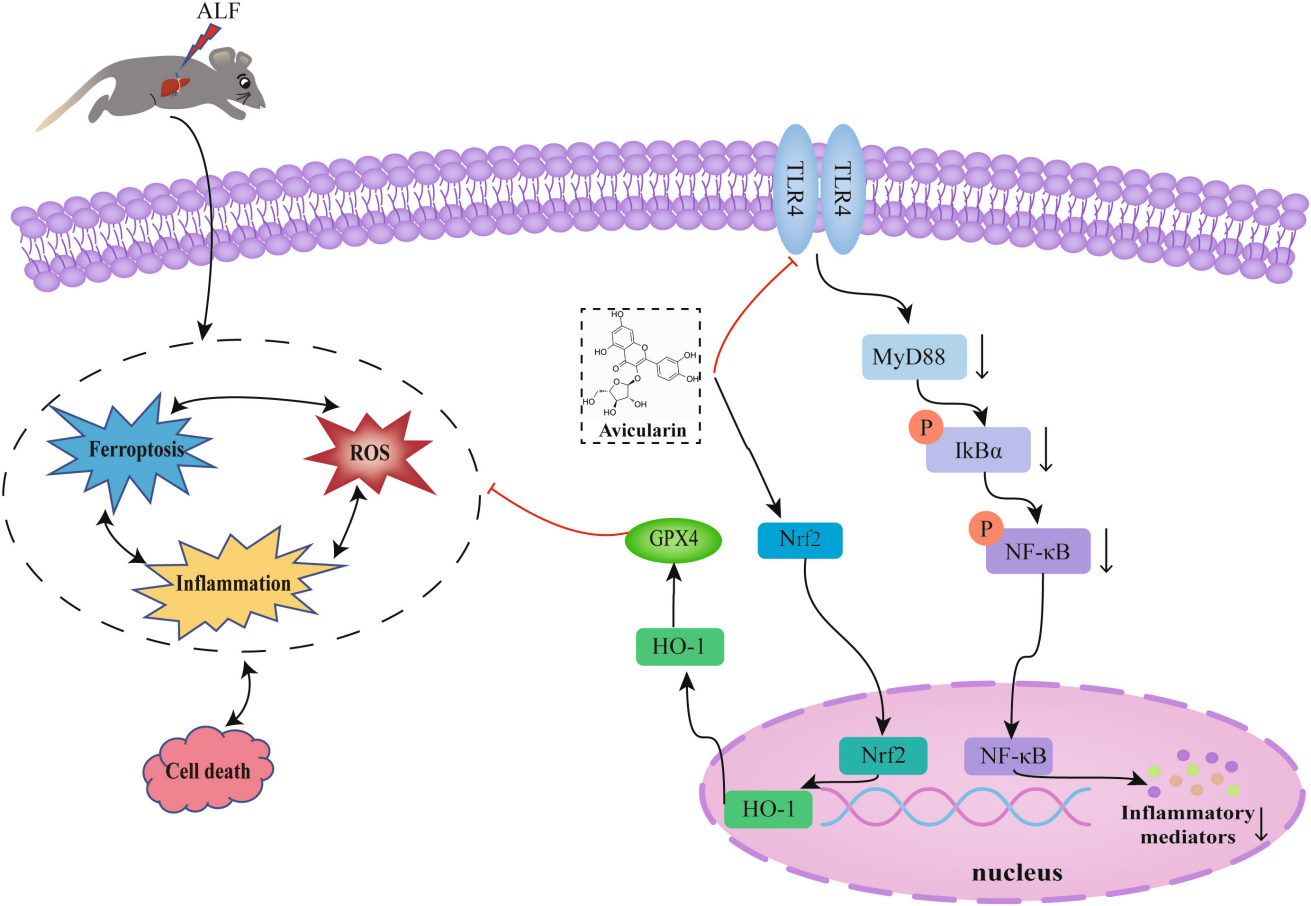
A schematic description of the effects of avicularin on acute liver failure. Avicularin can reduce the inflammatory response by inhibiting the TLR4/MyD88/NF‐κB pathway, suppress hepatocyte ferroptosis via activating the Nrf2/HO‐1/GPX4 pathway and ultimately alleviate LPS/ D‐GalN‐induced acute liver failure.

In conclusion, this study demonstrated that AL attenuates LPS/D‐GalN‐induced ALF by suppressing inflammation and ferroptosis through mechanisms that may be related to inhibition of TLR4/MyD88/NF‐κB and activation of Nrf‐2/HO‐1/GPX4 signalling pathways. This provides a novel potential pharmacological alternative for ALF. A schematic description of the effect and molecular mechanism of AL is expounded in Figure 9.

## AUTHOR CONTRIBUTIONS

**Pei Shi:** Investigation (equal); methodology (equal); writing – original draft (equal). **Wentao Zhu:** Conceptualization (equal); formal analysis (equal). **Jiwei Fu:** Methodology (equal); resources (equal). **An Liang:** Methodology (equal). **Ting Zheng:** Data curation (equal). **Zhilong Wen:** Resources (equal). **Xincheng Wu:** Formal analysis (equal). **Yuchen Peng:** Data curation (equal); methodology (equal). **Songsong Yuan:** Writing – review and editing (equal). **Xiaoping Wu:** Project administration (equal); writing – review and editing (equal).

## CONFLICT OF INTEREST STATEMENT

We declare that we have no financial and personal relationships with other people or organizations that could inappropriately influence this work. All authors have approved the submitted manuscript and confirmed that have no conflict of interest.

## Data Availability

Data will be available on request.
